# Andrographolide Exerts Antihyperglycemic Effect through Strengthening Intestinal Barrier Function and Increasing Microbial Composition of *Akkermansia muciniphila*

**DOI:** 10.1155/2020/6538930

**Published:** 2020-07-23

**Authors:** Hongming Su, Jianling Mo, Jingdan Ni, Huihui Ke, Tao Bao, Jiahong Xie, Yang Xu, Lianghua Xie, Wei Chen

**Affiliations:** ^1^Department of Food Science and Nutrition, National Engineering Laboratory of Intelligent Food Technology and Equipment, Zhejiang Key Laboratory for Agro-Food Processing, Zhejiang University, Hangzhou 310058, China; ^2^Department of Traditional Chinese Medicine, Sir Run Run Shaw Hospital, School of Medcine, Zhejiang University, Hangzhou 310016, China; ^3^Ningbo Research Institute, Zhejiang University, Ningbo 315100, China

## Abstract

Accumulating evidence indicates that type 2 diabetes (T2D) is associated with intestinal barrier dysfunction and dysbiosis, implying the potential targets for T2D therapeutics. Andrographolide was reported to have several beneficial effects on diabetes and its associated complications. However, the protective role of andrographolide, as well as its underlying mechanism against T2D, remains elusive. Herein, we reported that andrographolide enhanced intestinal barrier integrity in LPS-induced Caco-2 cells as indicated by the improvement of cell monolayer barrier permeability and upregulation of tight junction protein expression. In addition, andrographolide alleviated LPS-induced oxidative stress by preventing ROS and superoxide anion radical overproduction and reversing glutathione depletion. In line with the *in vitro* results, andrographolide reduced metabolic endotoxemia and strengthened gut barrier integrity in db/db diabetic mice. We also found that andrographolide appeared to ameliorate glucose intolerance and insulin resistance and attenuated diabetes-associated redox disturbance and inflammation. Furthermore, our results indicated that andrographolide modified gut microbiota composition as indicated by elevated Bacteroidetes/Firmicutes ratio, enriched microbial species of *Akkermansia muciniphila*, and increased SCFAs level. Taken together, this study demonstrated that andrographolide exerted a glucose-lowering effect through strengthening intestinal barrier function and increasing the microbial species of *A. muciniphila*, which illuminates a plausible approach to prevent T2D by regulating gut barrier integrity and shaping intestinal microbiota composition.

## 1. Introduction

Type 2 diabetes (T2D) is characterized by insulin resistance, glucose intolerance, and hyperglycemia [1]. The development and progression features of T2D among individuals can vary considerably as results of diverse genetic backgrounds and lifestyles. Although several antidiabetic treatment strategies have been recommended including pharmacological medications and lifestyle modifications (weight loss and exercise), the health benefits from these strategies are limited. Antidiabetic agents such as pioglitazone and acarbose are effective in preventing diabetes [2]. However, the potential side-effect of those antidiabetic agents cannot be overlooked [3]. In the face with a large number of T2D populations, it is urgently needed to develop novel, safe, and effective antidiabetic medications.

Gut microbiota has been recognized to be implicated with whole-body glucose and lipid homeostasis [4]. The connection between the microbial community and the human body is linked and separated by the intestinal barrier. The intestinal barrier function could be disrupted under obesity and diabetes conditions, which contribute to the increased gut permeability, leading to a more readily translocation of lipopolysaccharide (LPS) into the circulation [5]. LPS is confirmed to play a critical role in triggering systematic inflammation, which is also linked to the onset and development of insulin resistance [6]. Therefore, the intestinal epithelial barrier could be an alternative therapeutic target to prevent metabolic endotoxemia and thus provide metabolic benefits [7]. In addition, accumulating evidence implicates gut microbiota as a promising target for T2D therapeutics [8]. Recently, the species of *Akkermansia muciniphila*, a mucin-degradation and strictly anaerobic bacteria, has received considerable attention for its beneficial effect on metabolic disorders [9–11]. Multiple bioactive components such as metformin, cranberry extract, and dietary polyphenols are reported to confer metabolic benefits through changing the bacterial abundance of *A. muciniphila* [12–14]. Therefore, the investigation of potential candidate prebiotics, which could promote the growth of *A. muciniphila*, might provide possible strategies for the prevention and treatment of metabolic diseases including T2D.

Andrographolide is a diterpenoid lactone derived from *Andrographis paniculata* (Burm. F.) Nees [15]. This plant is widely distributed in Southeast Asia, such as China, India, and Thailand [16]. Andrographolide has been demonstrated to have a broad range of pharmacological activities such as anticancer, anti-inflammation, antiobesity, and anti-NAFLD [16–18]. Our previous studies have shown that andrographolide suppressed preadipocyte proliferation and inhibited hepatic carcinogenesis [15, 16]. Regarding the effect of andrographolide on diabetes, a previous study indicated the antihyperglycemic effect of andrographolide in streptozotocin- (STZ-) induced diabetic mice [19]. Andrographolide was also reported to attenuate postprandial hyperglycemia by inhibiting *α*-glucosidase and *α*-amylase enzyme activities [20]. However, whether andrographolide could enhance the gut epithelial barrier integrity or modulate the intestinal microbiota composition in diabetes remains unclear. Here, we established a colorectal cell monolayer barrier model to investigate the protective effect of andrographolide against LPS-induced disruption of monolayer barrier integrity and oxidative stress. Furthermore, the effect of andrographolide on gut barrier integrity and gut microbiota composition in a murine model of T2D was examined. Our results indicated that andrographolide prevented hyperglycemia through strengthening intestinal barrier function and increasing *A. muciniphila*.

## 2. Materials and Methods

### 2.1. Reagents

Dichlorodihydrofluorescein diacetate (DCFH-DA), dihydroethidium (DHE), lipopolysaccharide (LPS), and fluorescein-isothiocyanate- (FITC-) dextran (average MW 3,000-5,000) were purchased from Sigma-Aldrich (California, USA). Naphthalene-2,3-dicarboxal-dehyde (NDA) was obtained from Life Technologies (Carlsbad, CA, USA). SYBR Green PCR Master Mix was purchased from Roche (Basel, Switzerland). All other reagents used were of analytical grade.

### 2.2. Extraction and Purification of Andrographolide

The dry leaves of *A. paniculata* were mixed with 95% aqueous ethanol solution and then ultrasound extracted twice at 45°C for 1 h. Then, the ethanol extracts were combined, filtered, and concentrated. The crude mixture was reextracted with petroleum ether twice and followed by ethyl acetate extraction. The residue was obtained after the evaporation of ethyl acetate portion and used for high-speed countercurrent chromatography (HSCCC, TBE-300A, Tauto Biotechnique Company, Shanghai, China) separation and HPLC (Dionex Ultimate 3000, Thermo Fisher Scientific, USA) analysis [21]. The purity of andrographolide was 98.8%.

The structure of andrographolide was identified by NMR. Andrographolide was dissolved in 0.5 mL of CD_3_OD. The experiment was performed on a Bruker AVANCE™ III spectrometer (14.1 Tesla), with a Larmor frequency of 150 MHz for ^13^C and 600 MHz for ^1^H. ^1^H NMR of andrographolide (CD_3_OD, 600 MHz) is as follows: *δ* 6.86-6.88 (m, 1H), 5.03 (d, *J* = 6.0 Hz, 1H), 4.91 (d, *J* = 6.0 Hz, 1H), 4.69 (s, *J* = 6.0 Hz, 1H), 4.49 (dd, *J* = 10.2 Hz, 6.6 Hz, 1H), 4.18 (dd, *J* = 10.2 Hz, 1.8 Hz, 1H), 4.14 (d, *J* = 10.8 Hz, 1H), 3.38-3.44 (m, 2H), 2.57-2.68 (m, 2H), 2.43-2.47 (m, 1H), 2.03-2.08 (m, 1H), 1.94-1.96 (m, 1H), 1.84-1.89 (m, 2H), 1.80-1.83 (m, 2H), 1.36-1.43 (m, 1H), 1.30-1.35 (m, 2H), 1.24 (s, 3H), and 0.77 (s, 3H). ^13^C NMR of andrographolide (CD_3_OD, 150 MHz) is as follows: 172.6, 149.4, 148.8, 129.8, 109.2, 80.9, 76.1, 66.7, 65.0, 57.4, 56.3, 43.7, 40.0, 39.0, 38.1, 29.0, 25.7, 25.2, 23.4, and 15.5.

### 2.3. Cell Culture

Human Caco-2 cells were obtained from the Cell Bank of Type Culture Collection of Chinese Academy of Sciences (CBTCCCAS, Shanghai, China). Caco-2 cells were cultured in DMEM medium containing 10% fetal bovine serum, 100 IU/mL penicillin, and 100 *μ*g/mL streptomycin in a humidified cell incubator with an atmosphere of 5% CO_2_ at 37°C.

### 2.4. *In Vitro* Caco-2 Cell Monolayer Permeability Assay

Transepithelial electrical resistance (TEER) was determined using a Millicell-ERS-2 Volt-Ohm meter (Millipore) according to a previous report [22]. Briefly, Caco-2 cells (2 × 10^5^) were seeded onto Transwell plates (0.4 *μ*m pores; Corning, USA) for 21 days to reach confluence. After that, a Caco-2 monolayer grown on the apical side of Transwell plates was treated with andrographolide (2.5 and 5 *μ*M), followed by treatment of LPS (10 *μ*g/mL). The effect of andrographolide on the permeability of large molecular substances across the Caco-2 cell monolayer barrier was determined using FITC-dextran. After treatment, the medium in the basolateral and apical compartment was replaced with 1.5 mL DMEM or 0.5 mL DMEM containing FITC-dextran (1 mg/mL). After 2 h incubation, the concentration of FITC-dextran in the basolateral compartment was determined by a fluorescence microplate at a wavelength of 495 nm.

### 2.5. Fluorescence Microscopy

Reactive oxygen species (ROS), superoxide anion radicals (O_2_^−^), and glutathione (GSH) are determined according to previously described methods [23, 24]. Briefly, cells were seeded into 12-well plates a density of 6 × 10^4^ cells/well for 24 h. Cells were treated with andrographolide (2.5 and 5 *μ*M) for 24 h, followed by treating with LPS (10 *μ*g/mL) for further 24 h. After incubating with 10 *μ*M DCFH-DA (for ROS labeling) or 10 *μ*M DHE (for superoxide anion radical labeling) or 50 *μ*M NDA (for GSH labeling) for 30 min, cells were washed with PBS twice and then immediately evaluated by fluorescence microscopy. The results from the fluorescence microscope were expressed as mean fluorescence intensity. The fluorescence intensity was analyzed by Image-Pro Plus 6.0 (Media Cybernetics, Inc.).

### 2.6. Real-Time Reverse Transcription-PCR

We followed the methods of Su et al. [25]. Total RNA was isolated from the caecal tissue using TRIzol (Invitrogen, CA, USA) and then pooled for the RT-PCR analysis. cDNA was synthesized using the PrimeScript RT Reagent Kit (TaKaRa, Japan) according to the manufacturer's instruction. Quantitative real-time PCR was carried out in the QuantStudio 3 Real-Time PCR System (Applied Biosystems, CA, USA). The primers used in this study are shown in Table S1.

### 2.7. Western Blot

Western blot analysis was performed as previously described [15]. Total protein fraction was extracted using RIPA Lysis Buffer (BOSTER Biological Technology Co. Ltd.) with the addition of Roche cOmplete™ Protease Inhibitor Cocktail. Protein was separated by electrophoresis on SDS-polyacrylamide gels and transferred to polyvinylidene fluoride (PVDF) membranes (Millipore, ISEQ00010). After blocking with 10% nonfat dry milk in PBS buffer containing 0.1% Tween-20 (PBST), the membrane was incubated with the primary antibody overnight at 4°C. After three washes, the membrane was incubated with horseradish peroxidase-conjugated secondary antibodies (Bio-Rad, 170-6515 and 170-6516, 1 : 1000) for 1 h. After three washes with PBST, the immunoreactive protein bands were visualized by Chemiluminescent HRP Substrate (Millipore, WBKLS0100). The following primary antibodies were used: Occludin (Abcam, ab216327, 1 : 1000), ZO-1 (Abcam, ab61357, 1 : 1000), and GAPDH (Abcam, ab181602, 1 : 10,000).

### 2.8. *In Vivo* Experimental Design

All animal experiments were conducted according to the guidelines and laws on the use and care of laboratory animals in China (GB/T 35892-2018 and GB/T 35823-2018). The animal experimental procedures were performed in the Animal Experiment Center of Zhejiang Chinese Medical University (Hangzhou, China). The animal protocol was approved by the laboratory animal management and ethics committee of Zhejiang Chinese Medical University (201610087). Male Lepr^db^ mutation (db/db) mice with C57BL/6J background, aged six weeks, were purchased from the Model Animal Research Center of Nanjing University (Nanjing, China). All mice have *ad libitum* access to autoclaved water and diet. The temperature in the cage was maintained with constant temperature (23°C) and humidity. After one week of acclimatization, the mice were divided into two groups: (1) db/db mice were orally administered with vehicle (normal saline (NS)/Tween-80 (25 : 1, *v*/*v*) (*n* = 12); (2) db/db mice were orally administered with 150 mg/kg per mice andrographolide (AG) (*n* = 12), according to a previous report [26]. Andrographolide was dissolved in normal NS/Tween-80 (25 : 1, *v*/*v*) by grinding with a mortar and pestle. After eight weeks' administration, the mice were sacrificed under anesthesia using carbon dioxide, following 12 h fasting. Blood was collected by cardiac puncture and centrifuged at 5000 rpm for 10 min for serum collection. Caecal contents were collected immediately after euthanasia and preserved in sterilized tubes. Caecal contents, serum, and all of the tissues were snap-frozen and stored at -80°C.

### 2.9. Glucose Tolerance Test (GTT) and Insulin Tolerance Tests (ITT)

GTT and ITT were determined as previously described [25]. At week 7, mice fasted for 12 h, and GTT was performed after 0.5 g/kg glucose was administered intraperitoneally. Blood glucose levels were measured from the tail before glucose administration and 15, 30, 60, 90, and 120 min after administration. ITT was performed after mice fasted for 6 h. Insulin (2 IU/kg) was administered intraperitoneally. Blood glucose levels were measured from the tail before insulin administration and 15, 30, 60, 90, and 120 min after the administration.

### 2.10. Biochemical Analysis

The blood samples were collected and centrifuged at 5000 rpm for 10 min, and then serum was collected for biochemical analysis. Triglyceride (TG), cholesterol, free fatty acids (FFAs), aspartate transaminase (AST), alanine aminotransferase (ALT), lactate dehydrogenase (LDH), and glucose were measured by the Hitachi automatic biochemistry analyzer. Serum insulin, LPS, and LPS-binding protein (LBP) were determined using ELISA kits according to the manufacturer's instructions (Elabscience, Wuhan, China).

### 2.11. Homeostasis Model Assessment-Insulin Resistance (HOMA-IR)

The HOMA-IR index was calculated using the fasting values of glucose and insulin with the following formula: HOMA index = insulin (*μ*U/mL) × glucose (mM)/22.5.

### 2.12. Fecal Microbiota Identification

We followed the methods as previously described [25]. The genomic DNA from fecal samples was extracted using the QIAamp DNA Stool Mini Kit (Qiagen, Germany) according to the manufacturer's instruction. 16S rRNA comprising V3-V4 regions was amplified using 16S universal primers. Amplicons were purified using the AxyPrep DNA Gel Extraction Kit (Axygen Biosciences, CA, USA). The purified amplicons were sequenced on the Illumina MiSeq platform. Raw sequences were analyzed and processed using the Quantitative Insights into the Microbial Ecology (QIIME) software package. All sequences were clustered into Operational Taxonomic Units (OTUs) with a 97% threshold by using UPARSE. The taxonomy of each 16S rRNA gene sequence was analyzed by UPARSE mapping to Greengene (default): V201305 for species annotation. The relative abundance of each bacterial taxa was analyzed. Heatmaps were drawn using the ggplot2 package of the R software (v.3.1.1).

### 2.13. Short-Chain Fatty Acids (SCFAs) Identification and Quantification

SCFA levels in fecal content were analyzed using gas chromatography on Agilent 6890 (Agilent Technologies, CA, USA) in comparison with known standards as previously described [14].

### 2.14. Statistical Analysis

Data are expressed as the mean ± SEM. Two-tailed Student's *t*-test was performed to evaluate the significant differences between two groups. One-way analysis of variance (ANOVA) followed by Bonferroni's post hoc test was performed to evaluate the significant differences between multiple groups. Metastats analysis was used to determine the bacterial species with a statistically significant difference. *p* < 0.05 was considered to be a significant difference. The statistical analyses were performed using GraphPad Prism (V.7.0a, GraphPad Software, USA) and SPSS (Version 20.0).

## 3. Results

### 3.1. Andrographolide Attenuated LPS-Induced Disruption of Caco-2 Cell Monolayer Barrier Integrity

**Figure 1:**
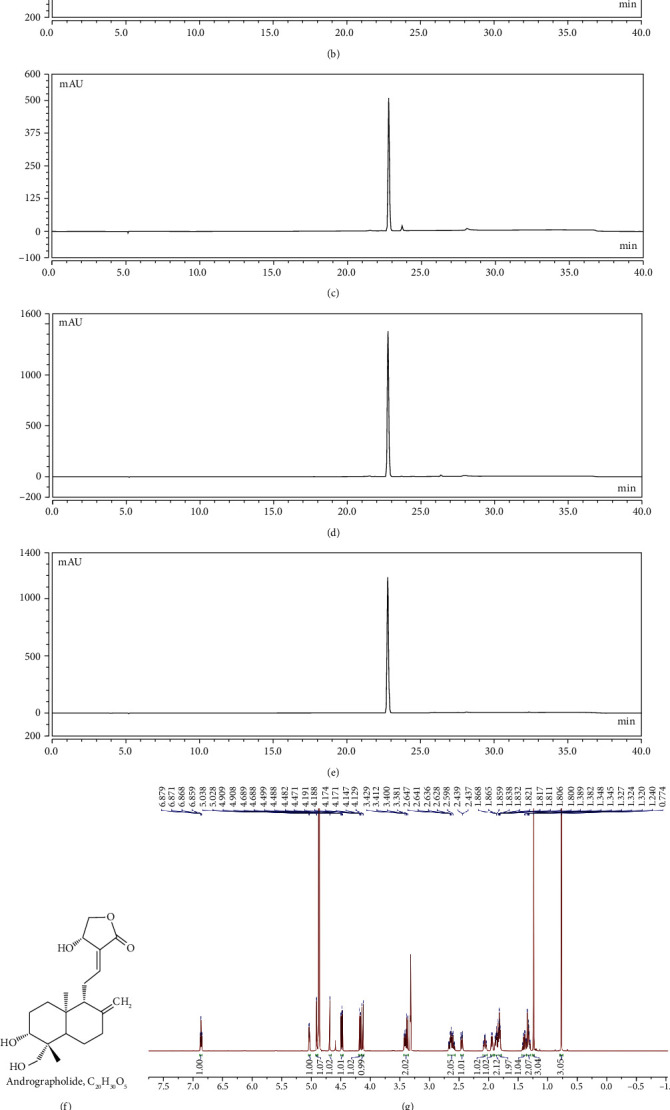
Purification and identification of andrographolide from *A. paniculata*. (a) The crude mixture was reextracted with petroleum ether twice and followed by ethyl acetate extraction. The ethyl acetate portion was analyzed by HPLC at 254 nm. (b) The fraction of 75-88 min in HSCCC separation was analyzed by HPLC at 254 nm. (c) The fraction of 75-88 min in HSCCC separation was evaporated and then washed with the solvent of ethyl acetate/methanol/water (1 : 2 : 4, *v*/*v*/*v*). (d) The andrographolide was isolated and was analyzed by HPLC at 254 nm. (e) HPLC analysis of the andrographolide of chromatographic grade at 254 nm. (f) Chemical structure of andrographolide. NMR spectrums of andrographolide, (g) ^1^H NMR of andrographolide, and (h) ^13^C NMR of andrographolide.

**Figure 2:**
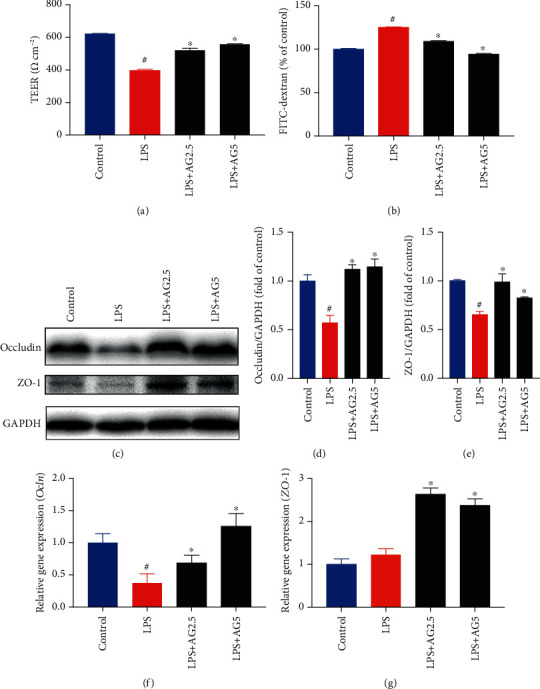
Andrographolide improved LPS-induced disruption of the intestinal barrier in Caco-2 cells. (a) Effect of andrographolide on TEER in LPS-induced Caco-2 cells. The Caco-2 cells were seeded in Transwell cell plates and cultured for 21 days to form a cell monolayer. Then, the cells were treated with andrographolide (2.5 and 5 *μ*M) for 24 h, followed by the treatment of LPS (10 *μ*g/mL) for a further 24 h. The TEER value was measured using a Millicell-ERS-2 Volt-Ohm meter (Millipore). (b) Effect of andrographolide on FITC-dextran concentration. The Caco-2 cells in Transwell cell plates were treated with andrographolide (2.5 and 5 *μ*M) for 24 h, followed by the treatment of LPS (10 *μ*g/mL) for a further 24 h. After treatment, the medium in the apical compartment was replaced with fresh DMEM containing FITC-dextran (1 mg/mL). After 2 h incubation, the medium from the basolateral compartment was subjected to spectrofluorometric measurement. (c–e) Immunoblot analysis of Occludin and ZO-1 protein expression from LPS-induced Caco-2 cells in the presence and absence of andrographolide (2.5 and 5 *μ*M) for 24 h. The protein bands were subjected to densitometric analysis using ImageJ. Relative mRNA expression of (f) *Ocln* and (g) *ZO-1* were determined by RT-PCR. Data represent means ± SEM (*n* = 3), ^#^*p* < 0.05 versus the control group, ∗*p* < 0.05 versus the LPS group.

Andrographolide was extracted and purified from the dry leaves of *A. paniculata* using high-speed countercurrent chromatography (HSCCC) and HPLC techniques, with a purity of 98.8% (Figures 1(a)–1(e)). The structure of andrographolide identified by NMR (Figures 1(f)–1(h)). The final yield of andrographolide in dry leaves of *A. paniculata* was 400 mg/kg. Accumulating evidence indicates that lipopolysaccharide (LPS), a principal component originated from bacteria, poses damage to gut barrier integrity [27]. Therefore, we employed the LPS-induced Caco-2 cell monolayer barrier model to investigate the gut barrier function involved in metabolic endotoxemia according to previous reports [22, 28]. To evaluate the protective role of andrographolide on cell monolayer permeability *in vitro*, the TEER and FITC-dextran concentration in the Transwell plates were determined. As shown in Figure 2(a), LPS (10 *μ*g/mL) treatment caused a significant reduction of TEER compared to the control, indicating the monolayer barrier permeability increased in Caco-2 cells upon LPS treatment. Andrographolide (2.5 *μ*M and 5 *μ*M) treatment significantly reversed LPS-induced TEER reduction compared with that in the LPS-treated group, indicating andrographolide improved monolayer barrier integrity. In line with the TEER results, we observed a significantly increased FITC-dextran concentration in LPS-induced cells, which indicated that large molecular substances were easier to pass through the LPS-induced Caco-2 cell monolayer barrier compared to the control (Figure 2(b)). However, andrographolide treatment significantly mitigated LPS-induced FITC-dextran fluorescence increase, indicating that andrographolide protected against LPS-induced intestinal monolayer barrier dysfunction. Since the monolayer barrier permeability was improved upon andrographolide treatment, we thus hypothesized that andrographolide reinforced intestinal barrier function through inducing tight junction protein expression. To test this possibility, we determined the effect of andrographolide on the expressions of tight junction protein including occluding (Ocln) and Zona occludin-1 (ZO-1). As anticipated, andrographolide treatment abolished LPS-induced reduction of occludin and ZO-1 protein expressions (Figures 2(c)–2(e)). Moreover, andrographolide treatment facilitated the upregulation of *Ocln* and *ZO-1* mRNA expressions compared with those in the LPS-induced cells (Figures 2(f) and 2(g)). Together, these results confirmed that andrographolide ameliorated LPS-induced disruption of cell monolayer permeability by inducing tight junction proteins.

### 3.2. Andrographolide Prevented LPS-Induced Oxidative Stress in Caco-2 Cells

**Figure 3:**
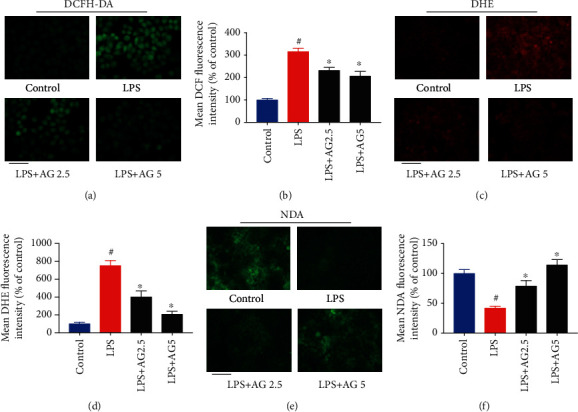
Andrographolide prevented LPS-induced oxidative stress. (a) Effect of andrographolide on LPS-induced ROS production. The Caco-2 cells were pretreated with andrographolide (2.5 and 5 *μ*M) for 24 h, followed by the treatment of LPS (10 *μ*g/mL) for a further 24 h. After that, the medium was removed and incubated with 10 *μ*M of DCFH-DA for 30 min, and then cells were evaluated by fluorescence microscopy. The scale bar represents 25 *μ*m. (b) The quantitative data of panel (a), and results were expressed as mean DCF fluorescence intensity (*n* = 6). (c) Effect of andrographolide on LPS-induced superoxide anion radical production. After treatment, cells were collected and incubated with 10 *μ*M of DHE for 30 min, and then cells were evaluated by fluorescence microscopy. The scale bar represents 50 *μ*m. (d) The quantitative data of panel (c), and results were expressed as mean DHE fluorescence intensity (*n* = 6). (e) Effect of andrographolide on GSH content in LPS-induced Caco-2 cells. After treatment, cells were collected and incubated with 50 *μ*M of NDA for 30 min, and then cells were evaluated by fluorescence microscopy. The scale bar represents 50 *μ*m. (f) The quantitative data of panel (e), and results were expressed as mean NDA fluorescence intensity (*n* = 6). Data represent means ± SEM (*n* = 6), ^#^*p* < 0.05 versus control group, ∗*p* < 0.05 versus the LPS group.

Increasing evidence indicates that metabolic endotoxemia is associated with oxidative stress [29, 30]. To uncover the protective effect of andrographolide against LPS-induced oxidative stress, we detected reactive oxygen species (ROS) using a fluorescence probe DCFH-DA. As shown in Figures 3(a) and 3(b), LPS (10 *μ*g/mL) treatment contributed to a significant increase of DCF fluorescence intensity compared with that in control, indicating an excessive production of ROS. Andrographolide treatment dramatically inhibited LPS-induced ROS overproduction in Caco-2 cells compared with the LPS-induced group, suggesting andrographolide is capable of preventing LPS-induced ROS production. As superoxide anion radical (O_2_^−^) is a particular type of ROS, which may lead to the generation of highly oxidizing derivative hydroxyl radical (·OH), we next determined whether andrographolide suppressed LPS-induced superoxide anion radical production. As shown in Figures 3(c) and 3(d), superoxide anion radical was measured by a DHE fluorescence probe. The results exhibited that LPS treatment contributed to a significant increase of DHE fluorescence intensity in comparison with that in control. However, andrographolide treatment markedly reduced the DHE fluorescence intensity compared with that in LPS-induced cells, indicating andrographolide inhibited LPS-induced superoxide anion radical production in Caco-2 cells. Intracellular GSH level as an essential indicator reflects cellular redox status. Therefore, we determined the effect of LPS on intracellular GSH level in the presence or absence of andrographolide. Cellular GSH level was monitored by a fluorescence probe NDA. As shown in Figures 3(e) and 3(f), LPS treatment resulted in a pronounced decrease of NDA fluorescence intensity compared with that in control. However, andrographolide treatment attenuated the reduction of NDA fluorescence intensity triggered by LPS, indicating andrographolide recovered LPS-induced GSH depletion. Collectively, our results suggest that andrographolide alleviated LPS-induced oxidative stress by inhibiting ROS, superoxide anion radical overproduction, and preventing GSH depletion.

### 3.3. Andrographolide Ameliorated Glucose Intolerance and Insulin Resistance by Enhancing Gut Barrier Integrity in db/db Mice

**Figure 4:**
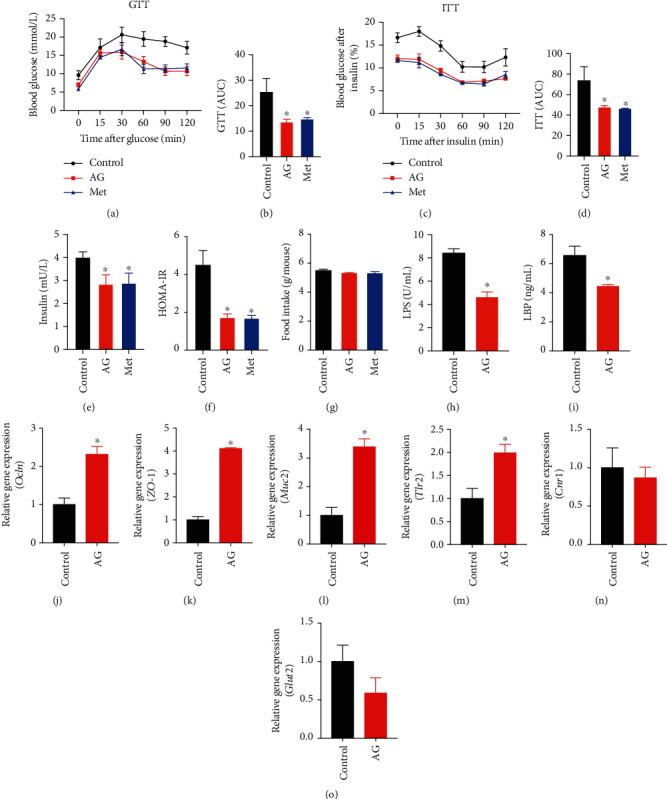
Andrographolide ameliorated glucose intolerance and insulin resistance by enhancing gut barrier integrity. 12 db/db mice were treated daily with vehicle, and 12 db/db mice were treated daily with andrographolide (150 mg/kg per day) or metformin (200 mg/kg per day) for 8 weeks by oral gavage. (a) At the eighth week, glucose tolerance test (GTT) was performed. (b) The area under the curve (AUC) of GTT was calculated. (c) Insulin tolerance test was performed at the end of the eighth week. (d) The AUC of ITT was calculated. (e) The serum insulin level and (f) HOMA-IR index were determined. (h) Serum LPS and (i) LBP were determined by the ELISA assay. The relative gene expression of (j) *Ocln*, (k) *ZO-1*, (l) *Muc2*, (m) *Tlr2*, (n) *Cnr1*, and (o) *Glut2* were determined. Control: db/db mice administered with vehicle; AG: db/db mice administered with 150 mg/kg andrographolide; Met: db/db mice administered with 200 mg/kg metformin. Data represent means ± SEM (*n* = 12), ∗*p* < 0.05 versus the control group.

Numerous studies have established the role of metabolic endotoxemia in the development of type 2 diabetes (T2D) [6], and the gut barrier integrity appeared to be disturbed in diabetic condition [31, 32]. To elucidate the protective role of andrographolide against T2D, we performed the glucose tolerance test (GTT) and insulin tolerance test (ITT) in db/db mice orally treated with andrographolide (150 mg/kg per mice) or vehicle for 8 weeks. Metformin, a widely used antidiabetic agent, was used as a positive control. The area under the curve (AUC) of GTT and ITT was calculated. Our results showed that andrographolide significantly improved glucose tolerance and insulin tolerance in db/db diabetic mice compared with those in control (Figures 4(a)–4(d)), which was similar to the results of metformin treatment (200 mg/kg per mice). In addition, fasting insulin level and HOMA-IR index significantly reduced after 8 weeks of andrographolide treatment (Figures 4(e) and 4(f)), which was in accordance with metformin treatment, suggesting andrographolide prevents insulin resistance in diabetic mice. However, andrographolide treatment did not influence daily food intake between groups (Figure 4(g)), indicating andrographolide did not change the appetite of mice. These results suggest that andrographolide ameliorates glucose intolerance and insulin resistance in db/db diabetic mice.

Since the *in vitro* results showed that andrographolide attenuated LPS-induced monolayer barrier dysfunction in Caco-2 cells, we speculated that andrographolide could improve intestinal barrier function in db/db mice. To examine the effect of andrographolide on metabolic endotoxemia, we determined the serum LPS and LPS-binding protein (LBP) levels. As shown in Figures 4(h) and 4(i), andrographolide administration contributed to a significant increase of LPS and LBP levels compared to the control, indicating the reduction of endotoxemia. To ascertain the effect of andrographolide on gut barrier integrity, we determined the relative gene expression of *Ocln* and *ZO-1*. Andrographolide administration led to the upregulation of *Ocln* and *ZO-1* mRNA expression compared with those in control (Figures 4(j) and 4(k)), indicating andrographolide improved the gut barrier integrity. Given that mucin 2 (*Muc2*) have a beneficial role in the gut barrier [33], we determined *Muc2* gene expression. Our study displayed that andrographolide significantly increased *Muc2* gene expression compared with that in control (Figure 4(l)). Toll-like receptor 2 (*Tlr2*) was reported to regulate tight junction proteins [34]. Hence, we investigated whether andrographolide could activate *Tlr2*. As shown in Figure 4(m), *Tlr2* expression was significantly increased upon andrographolide administration compared to the control. In addition, cannabinoid receptor 1 (*Cnr1*) activation was confirmed to increase intestinal permeability, whereas blocking *Cnr1* reduced gut permeability [35]. However, in the present study, the expression of *Cnr1* was not influenced by andrographolide administration (Figure 4(n)). To further investigate the protective role of andrographolide on intestinal barrier function, we determined the intestinal expression of glucose transporter 2 (*Glut2*), as a recent study reported that hyperglycemia drives the intestinal barrier dysfunction through Glut2-dependent mechanism [31]. We observed that *Glut2* gene expression was slightly decreased with no significant difference in andrographolide-treated mice compared to the control (Figure 4(o)). Altogether, these data suggest that andrographolide enhanced gut barrier function might through activating Tlr2.

### 3.4. Andrographolide Improved Serum Biochemical Profiles

To investigate the effect of andrographolide on serum biochemical profile, we determined serum triglyceride (TG) (Figure S1 (A)), cholesterol (Figure S1(B)), and free fatty acids (FFAs) (Figure S1 (C)). After 8 weeks' andrographolide treatment, serum TG, cholesterol, and FFAs significantly reduced on db/db diabetic mice compared with those in control. In addition, andrographolide administration contributed to a significant decrease of AST (Figure S1 (D)), ALT (Figure S1 (E)), and LDH (Figure S1 (F)) compared with those in control, suggesting andrographolide could improve hepatic function. These results indicate andrographolide improves serum biochemical profiles in db/db mice.

### 3.5. Andrographolide Improved Intestinal Redox Status and Inflammatory Response

**Figure 5:**
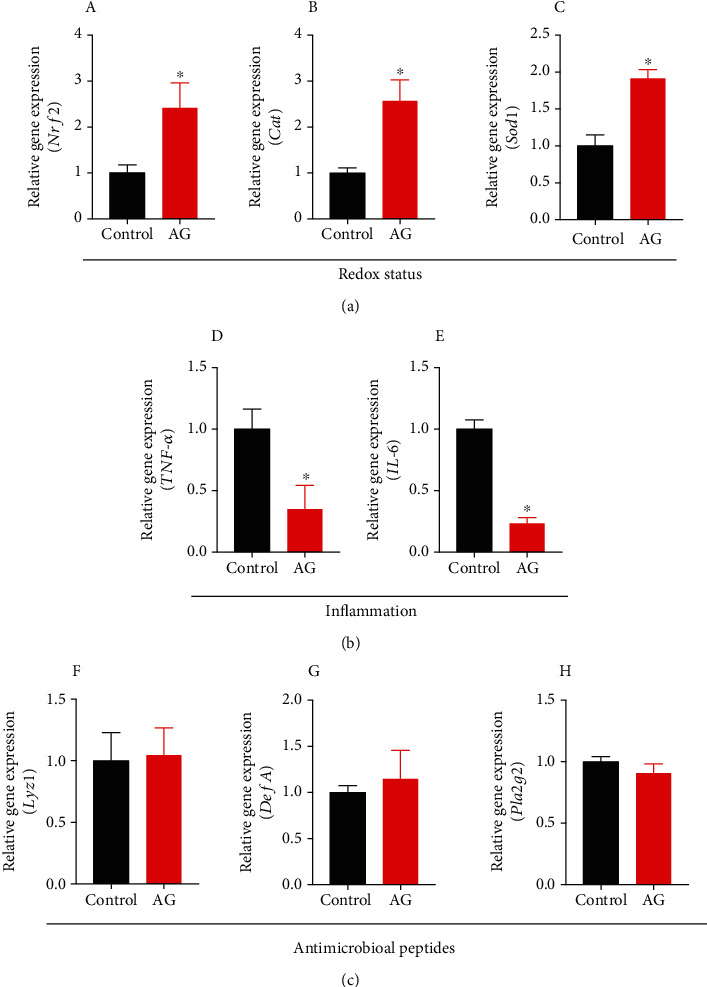
Andrographolide attenuated diabetes-associated redox disturbance and inflammation. (a) The relative gene expression from caecal tissue corresponding to redox status (A) *Nrf2*, (B) *Cat*, and (C) *Sod1* was determined. (b) The relative gene expression of inflammatory factors from caecal tissue including (D) *TNF-α* and (E) *IL-6* were determined. (c) The relative gene expression of the antimicrobial peptides from caecal tissue including (F) *Lyz1*, (G) *DefA*, and (H) *Pla2g2* was determined. Control: db/db mice administered with vehicle; AG: db/db mice administered with 150 mg/kg andrographolide. Data represent means ± SEM (*n* = 6), ∗*p* < 0.05 versus the control group.

To further investigate the effect of andrographolide on intestinal redox status, we determined the relative gene expressions of nuclear factor- (erythroid-derived 2) like 2 (*Nrf2*), catalase (*Cat*), and superoxide dismutase 1 (*Sod1*), which play critical roles in maintaining redox balance. We found that andrographolide significantly increased the relative gene expressions of *Nrf2*, *Cat*, and *Sod1* compared with those in control (Figures 5(a)–5(c)), indicating andrographolide attenuates diabetes-associated oxidative stress. To examine the role of andrographolide on intestinal epithelial inflammation, we determined the relative gene expressions of tumor necrosis factor-*α* (TNF-*α*) and interleukin 6 (IL-6). Our study showed that the gene expressions of TNF-*α* and IL-6 were significantly decreased by andrographolide treatment (Figures 5(d) and 5(e)). Given that antimicrobial peptides play a crucial role in maintaining intestinal barrier function, redox status, and inflammation [36], we determined *Lyz1* (encoding for Lysosome-1), *DefA* (encoding defensin), and *Pla2g2* (encoding phospholipase A2 group-II) expressions (Figures 5(f)–5(h)). However, andrographolide had no significant impact on *Lyz1*, *DefA*, and *Pla2g2* gene expressions. Together, our study suggests that andrographolide improves diabetes-associated intestinal redox imbalance and inflammation without affecting antimicrobial peptides expression.

### 3.6. Andrographolide Modified the Gut Microbiota Composition and Increased the Bacterial Species of a. Muciniphila

**Figure 6:**
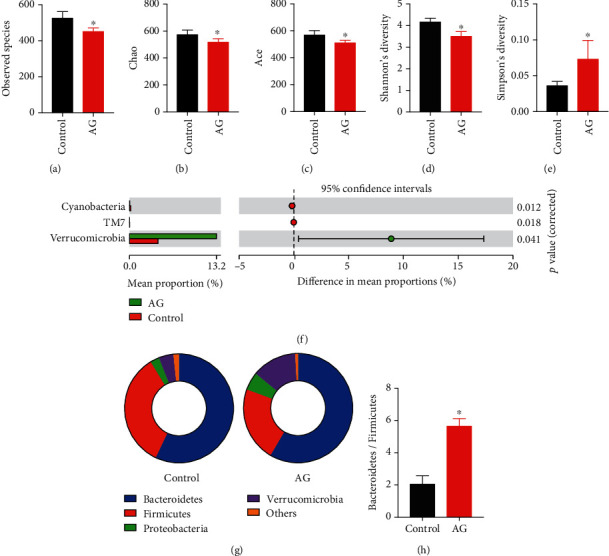
Andrographolide modulated gut microbiota composition. (a) Observed species, (b) Chao, (c) Ace, (d) Shannon's diversity, and (e) Simpson's diversity were obtained by analyzing 16S rDNA sequencing. (f) Statistical comparisons of gut bacterial profiles at the phylum level. (g, h) The Bacteroidetes/Firmicutes ratio was calculated. Control: db/db mice administered with vehicle; AG: db/db mice administered with 150 mg/kg andrographolide. Data represent means ± SEM (*n* = 6), ∗*p* < 0.05 versus the control group.

Gut microbiota can directly contact with the intestinal barrier and thereby exert its beneficial or detrimental impact on host health [7]. Although our study has shown that andrographolide improved gut barrier function both *in vivo* and *in vitro*, we cannot exclude the potential action of gut microbiota on host metabolic health. To further elucidate the potential involvement of gut microbiota on diabetes, we identified the caecal microbial composition using 16S rRNA sequencing. Beta-diversity analysis indicated that the observed species in the andrographolide-treated group was lower than that in the control group, suggesting a reduced enrichment of gut microbiota (Figure 6(a)). In addition, andrographolide caused reduced parameters of Chao, Ace, and Shannon's diversity compared with those in control (Figures 6(b)–6(d)). Simpson's diversity in andrographolide-treated group was higher than that in the control group (Figure 6(e)). To ascertain the enrichment alteration of specific gut microbiota in each taxa level, the OTUs were annotated, and their relative abundance was computed. As shown in Figure 6(f), andrographolide treatment resulted in a significant enrichment of *Verrucomicrobia* at the phylum level, whereas *TM7* and *Cyanobacteria* were significantly reduced compared with those in control. The increased abundance of Bacteroidetes/Firmicutes was associated with a beneficial effect on glucose metabolism [37]. In the present study, we observed an increased ratio of Bacteroidetes/Firmicutes (Figures 6(g) and 6(h)), suggesting the beneficial effect andrographolide on modulating gut microbiota composition. The heatmap shown in Figure S2 indicates the overall enrichment of gut microbiota in each group.

**Figure 7:**
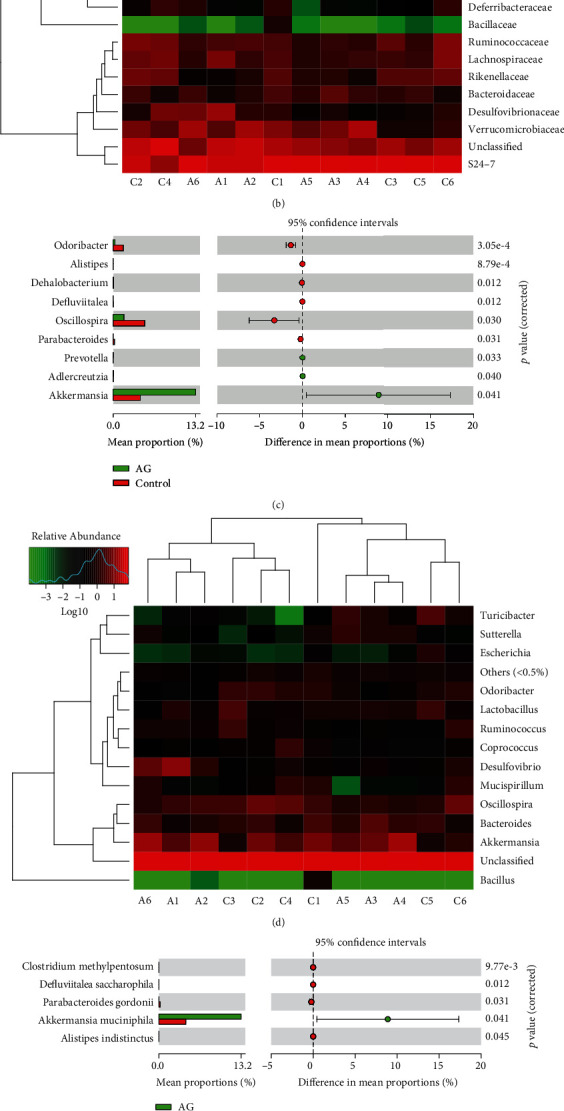
Andrographolide promoted the growth of *Akkermansia muciniphila*. (a) Statistical comparisons of gut bacterial profiles at the family level. (b) Heatmap of gut bacterial profiles at the family level. (c) Statistical comparisons of gut bacterial profiles at the genus level. (d) Heatmap of gut bacterial profiles at the genus level. (e) Statistical comparisons of gut bacterial profiles at the species level. (f) Heatmap of gut bacterial profiles at the species level. Control: db/db mice administered with vehicle; AG: db/db mice administered with 150 mg/kg andrographolide. Data represent means ± SEM (*n* = 6), ∗*p* < 0.05 versus the control group.

To further distinguish the differences of microbial community between the control and andrographolide-treated mice, the gut microbiota composition at family, genus, and species levels was specified. As shown in Figures 7(a) and 7(b), andrographolide treatment contributed to a significant enrichment of Verrucomicrobiaceae, Porphyromonadaceae, Coriobacteriaceae, and Erysipelotrichaceae at the family level, whereas Rikenellaceae, Odiribacteraceae, and Ruminococcaceae reduced. At the genus level, andrographolide increased the levels of *Akkermansia*, *Prevotella*, and *Adlercreutzia* and decreased the levels of *Odoribacter*, *Alistipes*, *Dehalobacterium*, *Defluviitalae*, *Oscillospira*, and *Parabacteroides* (Figures 7(c) and 7(d)). Interestingly, we observed that a beneficial bacterial species of *A. muciniphila* was significantly enriched in andrographolide-treated mice (Figures 7(e) and 7(f)). Together, these results suggest that andrographolide might provide metabolic benefits by shaping the microbiota composition and promoting the growth of *A. muciniphila*.

### 3.7. Andrographolide Increased Fecal Short-Chain Fatty Acids (SCFAs)

**Figure 8:**
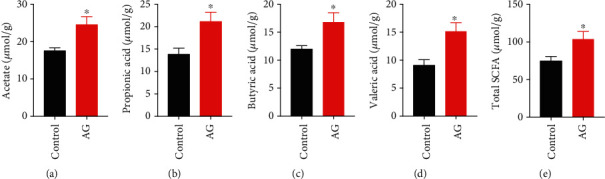
Andrographolide improved the short-chain fatty acids (SCFAs) composition. (a) acetate, (b) propionic acid, (c) butyric acid, and (d) valeric acid were measured in fecal samples by gas chromatography (GC) in comparison with known standards. (e) Total SCFAs were the sum of each amount of individual fatty acids. Control: db/db mice administered with vehicle; AG: db/db mice administered with 150 mg/kg andrographolide. Data represent means ± SEM (*n* = 6), ∗*p* < 0.05 versus the control group.

SCFAs have been proposed to play a critical role on the maintenance of the intestinal barrier [38]. Therefore, we determined the fecal concentration of SCFAs, including acetic acid, propionic acid, butyric acid, and n-valeric acid by gas chromatography. As expected, we found that andrographolide significantly increased the fecal concentration of total SCFAs, particularly acetic acid, propionic acid, and butyric acid in diabetic mice (Figures 8(a)–8(e)). Those results suggest an involvement of SCFAs on the protective action of andrographolide against leaky gut.

## 4. Discussion

Previous studies have confirmed the usefulness of andrographolide in preventing diabetes and its associated metabolic disorders through regulating multiple pathways. For example, andrographolide was reported to prevent diabetes-associated cognitive deficits [39]. In addition, andrographolide not only ameliorated diabetic nephropathy by inhibiting hyperglycemia-induced renal oxidative stress and inflammation via the Akt/NF-*κ*B pathway [40] but also attenuated diabetic retinopathy by inhibiting retinal angiogenesis and inflammation [41]. Andrographolide derived from *Andrographis paniculata* (Burm. F.) Nees exhibited profound antidiabetic promise [42], suggesting its potential preclinical and clinical application on preventing diabetes. Although the hypoglycemic effect of andrographolide has been reported long before [19, 43], its underlying mechanism is still largely unknown. Yu et al. indicated that andrographolide reduced the plasma glucose level in streptozotocin- (STZ-) induced diabetic mice through an increase of glucose utilization. The increase of GLUT4 gene expression was considered as one of the mechanisms of andrographolide [19]. Zhang et al. indicated that the andrographolide-lipoic acid conjugate (AL-1) prevented diabetes in alloxan-treated diabetic mouse model by protecting beta cell mass, preserving insulin-secreting function, and stimulating GLUT4 translocation [43]. Moreover, andrographolide was reported to prevent postprandial hyperglycemia by inhibiting *α*-glucosidase activity [20]. In the present study, we unveiled a novel mechanism that andrographolide ameliorated insulin resistance and glucose intolerance through reinforcement of the intestinal barrier function and modulation of the gut microbiota composition.

The intestinal barrier plays a critical role in making digested food components selectively absorbed. The increased intestinal barrier permeability may lead to a more readily permeation of xenobiotics or microbe-derived small molecules such as LPS [25, 27]. The increase of circulating LPS results in systemic inflammation and insulin resistance [6]. Accordingly, the gut barrier plays pivotal roles in maintaining host metabolic health. Tight junction proteins including occludin and ZO-1 constitute the wall between gut epithelial cells. Previous studies demonstrated that deficiency of tight junction protein might increase intestinal permeability [44], which led to the translocation of LPS into blood, suggesting an important connection between tight junction protein and inflammation. Andrographolide derivative was found to ameliorate dextran sulfate sodium-induced colitis by suppressing inflammation in mice [45], suggesting the potential protective role of andrographolide on gut barrier. However, little information is available concerning the direct role of andrographolide on intestinal barrier permeability.

In the present study, our results revealed that andrographolide restored LPS-induced disruption of monolayer barrier permeability *in vitro* as indicated by increased TEER and reduced FITC-dextran concentration. In addition, this study demonstrated that andrographolide strengthened the intestinal barrier integrity by inducing occludin and ZO-1. In consistence with the *in vitro* results, andrographolide displayed a profound protective role in enhancing gut barrier function by increasing the intestinal gene expression of occludin and ZO-1 in diabetic mice. Antimicrobial peptides produced by the host play crucial roles in maintaining gut microbiota homeostasis and gut barrier function [36]. However, andrographolide had no impact on *Lyz1*, *DefA*, and *Pla2g2* expressions, suggesting antimicrobial peptides were not involved in the protective action of andrographolide on gut barrier integrity. SCFAs including acetate, butyrate, and propionate, which are produced by bacterial fermentation, are the dominant SCFAs in the large intestine [27, 36]. SCFAs mediated several beneficial effects on host metabolic health. The reduction of SCFAs is associated with weakened tight junctions and permeability [46]. Andrographolide treatment increased total SCFAs, particularly acetic acid, propionic acid, butyric acid, and valeric acid levels, suggesting SCFAs are involved in andrographolide's beneficial effects on gut barrier function. Collectively, our results suggest that andrographolide provides metabolic benefits partially through the enhancement of intestinal barrier function by inducing tight junction proteins and promoting SCFAs.

Previous studies reveal that endotoxemia triggers cellular oxidative stress. The disrupted redox balance is responsible for the initiation and progression of inflammation [29, 30]. Andrographolide was found to inhibit TNF-*α*-induced ROS generation and GSH content depletion [24]. We demonstrated that andrographolide prevented LPS-induced oxidative stress by scavenging excessive ROS and superoxide anion radicals, as well as restoring GSH depletion in Caco-2 cells. A large number of evidences confirmed the critical role of Nrf2, a transcriptional factor regulating cellular redox homeostasis [47]. Activation of Nrf2 contributes to the upregulation of its target genes involved in the antioxidant signaling pathway. *In vivo* study confirmed that andrographolide promoted the expressions of *Nrf2*, *Cat*, and *Sod1*, suggesting andrographolide confers antioxidant defense against diabetes-associated oxidative stress. In addition, andrographolide alleviated inflammatory responses as indicated by the downregulation of IL-6 and TNF-*α*. It is possible that the decreased oxidative stress and inflammation might be extrapolated from the reinforced intestinal barrier by andrographolide.

Numerous studies indicate the association of intestinal microbiota with T2D [25, 48]. Structural modulation of gut microbiota is a putative strategy for the alleviation of T2D. In the present study, we performed Illumina sequencing of the V3 and V4 regions of the 16S rRNA gene and identified the taxonomic composition of caecal microbiota from andrographolide and vehicle-treated mice. We found the gut microbiota of andrographolide-treated mice were strikingly distinct from the control mice, implying the possible involvement of gut microbiota in preventing diabetes. Increasing evidence confirmed that increased abundance of Bacteroidetes/Firmicutes was associated with decreased glucose level and reduced insulin resistance [37]. As anticipated, andrographolide treatment exhibited an increased ratio of Bacteroidetes/Firmicutes. Of note, we found a microbial species of *A. muciniphila* was significantly enriched in andrographolide-treated mice. *A. muciniphila*, a Gram-positive strictly anaerobic bacterium, resides in intestinal mucosa showed great capability on the maintenance of host lipid and glucose homeostasis [9]. Recently, several natural products that promote the growth of *A. muciniphila* have been found, such as metformin, cranberry extract, and grape polyphenols [12–14]. The purified membrane proteins derived from *A. muciniphila* exerted its beneficial effects on diabetes and obesity [33], suggesting that *A. muciniphila* could be a potential bacterial target for antidiabetic drug development. Interestingly, in the present study, we also observed the phenomena that andrographolide increased the abundance of *A. muciniphila* in diabetic mice. Therefore, these results reveal that andrographolide prevents T2D in db/db mice presumably through increasing the Bacteroidetes/Firmicutes ratio and promoting the abundance *A. muciniphila*.

## 5. Conclusion

**Figure 9:**
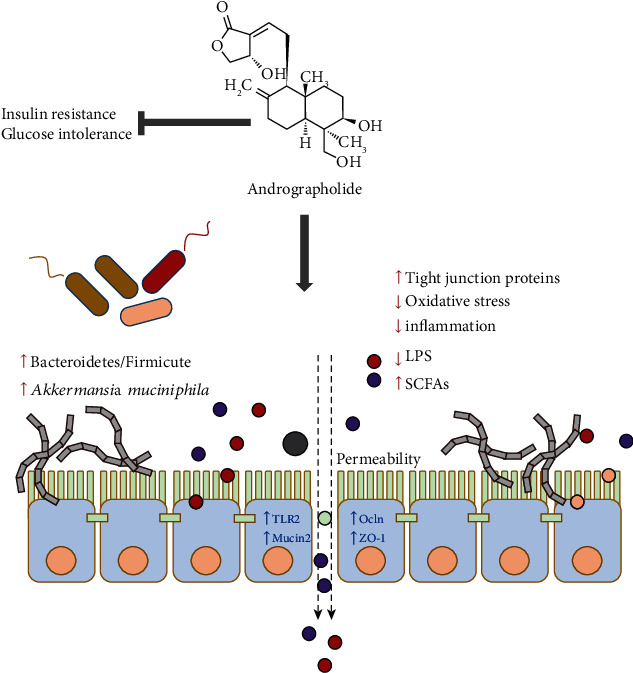
Brief summary of the mechanism of andrographolide on diabetes. This study demonstrated that andrographolide ameliorated glucose intolerance and insulin resistance in db/db diabetic mice. Andrographolide exerted glucose-lowering effect through strengthening intestinal barrier function and increasing microbial composition of *A. muciniphila*, which illuminates a plausible approach to ameliorate T2D.

In the present study, we aimed at investigating the protective effect of andrographolide derived from *A. paniculata* against T2D through regulating gut barrier integrity and gut microbiota composition. This study uncovers that andrographolide appears to attenuate insulin resistance and glucose intolerance in db/db mice through enhancing gut barrier integrity, elevating Bacteroidetes/Firmicutes ratio, and promoting the species abundance of *A. muciniphila*. In addition, andrographolide improved diabetes-associated redox disturbance and inflammation. The antidiabetic action of the mechanism of andrographolide is summarized in Figure 9. Those results illuminate a plausible approach to prevent T2D through regulating gut barrier integrity and modulating gut microbiota composition, which shed a novel insight that andrographolide may represent a promising agent in the prevention and treatment of T2D. Our results also suggest beneficial roles of andrographolide against oxidative stress and intestinal dysbiosis.

## Data Availability

The data used to support the findings of this study are available from the corresponding author upon request.

## Abbreviations

T2D: Type 2 diabetes
AG: Andrographolide
HSCCC: High-speed counter-current chromatography
TEER: Transepithelial electrical resistance
FITC-dextran: Fluorescein-isothiocyanate-dextran
Ocln: Occludin
ZO-1: Zonula occludens-1
ROS: Reactive oxygen species
DCFH-DA: Dichlorodihydrofluorescein diacetate
DHE: Dihydroethidium
NDA: Naphthalene-2,3-dicarboxal-dehyde
GSH: Glutathione
GTT: Glucose tolerance test
ITT: Insulin tolerance test
AUC: Area under the curve
HOMA-IR: Homeostasis model assessment of insulin resistance
LPS: Lipopolysaccharide
LBP: LPS-binding protein
Muc2: Mucin 2
Tlr2: Toll-like receptor 2
Cnr1: Cannabinoid receptor 1
Glut2: Glucose transporter 2
FFAs: Free fatty acids
AST: Aspartate transaminase
ALT: Alanine aminotransferase
LDH: Lactate dehydrogenase
Nrf2: Nuclear factor- (erythroid-derived 2) like 2
Cat: Catalase
Sod1: Superoxide dismutase 1
SCFAs: Short-chain fatty acids
TNF-*α*: Tumor necrosis factor-*α*
IL-6: Interleukin-6
Lyz1: Lysosome-1
DefA: Defensin A
Pla2g2: Phospholipase A2 group-II.

## Supplementary Materials

Supplementary materials Table S1: primers used for quantitative RT-PCR. Figure S1: andrographolide improved serum biochemical profiles. (A) Serum triglyceride, (B) cholesterol, (C) FFAs, (D) AST, (E) ALT, and (F) LDH levels were determined. Control: db/db mice administered with vehicle; AG: db/db mice administered with 150 mg/kg andrographolide. Data represent means ± SEM (*n* = 12), ∗*p* < 0.05 versus the control group. Figure S2: heatmap of gut bacterial profiles. OUT annotation of gut microbiota with significant difference between control (C) and andrographolide (A) groups. Control: db/db mice administered with vehicle; AG: db/db mice administered with 150 mg/kg andrographolide, *n* = 6, ∗*p* < 0.05 versus the control group.
