# Supplementary material for: Andrographolide Exerts Antihyperglycemic Effect through Strengthening Intestinal Barrier Function and Increasing Microbial Composition of Akkermansia muciniphila
Source: Oxid Med Cell Longev. 2020 Jul 23;2020:6538930. doi: 10.1155/2020/6538930 (PMC7396114; doi:10.1155/2020/6538930)
Supplement: Supplementary materials — Table S1: primers used for quantitative RT-PCR. Figure S1: andrographolide improved serum biochemical profiles. (A) Serum triglyceride, (B) cholesterol, (C) FFAs, (D) AST, (E) ALT, and (F) LDH levels were determined. Control: db/db mice administered with vehicle; AG: db/db mice administered with 150 mg/kg andrographolide. Data represent means ± SEM (n = 12), ∗p < 0.05 versus the control group. Figure S2: heatmap of gut bacterial profiles. OUT annotation of gut microbiota with significant difference between control (C) and andrographolide (A) groups. Control: db/db mice administered with vehicle; AG: db/db mice administered with 150 mg/kg andrographolide, n = 6, ∗p < 0.05 versus the control group. [file 6538930.f1.pdf]

Supplementary information

Andrographolide exerts antihyperglycemic effect through strengthening intestinal barrier function and increasing microbial composition of *Akkermansia muciniphila*

Hongming Su, Huihui Ke, Tao Bao, Jiahong Xie, Yang Xu, Lianghua Xie, Wei Chen*

Department of Food Science and Nutrition, Zhejiang University, Hangzhou 310058, China.

Supplementary Table 1. Primers used for quantitative RT-PCR.

Primer	Forward	Reverse
ZO-1 (H)	CTCCTGGATTTGGATTTGGA	CTGTCCTTCAGCTGGTCCTC
Ocln (H)	ATGGCTGCCTTTTGTTTCAT	ATGCCCAGGATAGCACTCAC
ZO-1 (M)	AGGACACCAAAGCATGTGAG	GGCATTCCTGCTGGTTACA
Tlr2 (M)	GCCTTTCAGGGAATTAAGCTCC	GATCAACCGATGGACGTGTAAA
Cnr1 (M)	CTGATGTTCTGGATCGGAGTC	TCTGAGGTGTGAATGATGATGC
Glut2 (M)	ACTTGGAAGGATCAAAGCAATGT	CAGTCCTGAAATTAGCCCACAA
Nrf2 (M)	TAGATGACCATGAGTCGCTTGC	GCCAAACTTGCTCCATGTCC
Cat (M)	CCAGCGACCAGATGAAGCAG	CCACTCTCTCAGGAATCCGC
Sod1 (M)	CAGCATGGGTTCCACGTCCA	CACATTGGCCACACCGTCCT
TNF-α (M)	AGGCACTCCCCCAAAAGAT	CAGTAGACAGAAGAGCGTGGTG
IL-6 (M)	CTTCCATCCAGTTGCCTTCTTG	AATTAAGCCTCCGACTTGTGAAG
Ocln (M)	ATGTCCGGCCGATGCTCTC	TTTGGCTGCTCTTGGGTCTGTAT
Lyz1 (M)	GCCAAGGTCTACAATCGTTGTGAGTTG	CAGTCAGCCAGCTTGACACCACG
Pla2g2 (M)	AGGATTCCCCCAAGGATGCCAC	CAGCCGTTTCTGACAGGAGTTCTGG
DefA (M)	GGTGATCATCAGACCCCAGCATCA	AAGAGACTAAAACTGAGGAGCAGC
Muc2 (M)	GCTCGGAACTCCAGAAAGAAG	GCCAGGGAATCGGTAGACAT
GAPDH (M)	AGGTCGGTGTGAACGGATTTG	TGTAGACCATGTAGTTGAGGTCA
GAPDH (H)	CTGGGCTACACTGAGCACC	AAGTGGTCGTTGAGGGCAATG

Supplementary Fig. S1

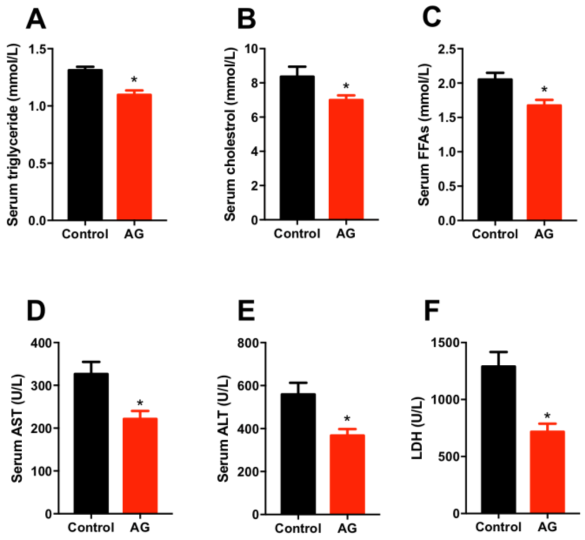

Supplementary Figure S1. Andrographolide improved serum biochemical profiles. (A) serum triglyceride, (B) cholesterol, (C) FFAs, (D) AST, (E) ALT and (F) LDH levels were determined. Control, db/db mice administered with vehicle; AG, db/db mice administered with 150 mg/kg andrographolide. Data represent means ± s.e.m. (n=12), *$p < 0.05$ versus control group.

Supplementary Fig. S2

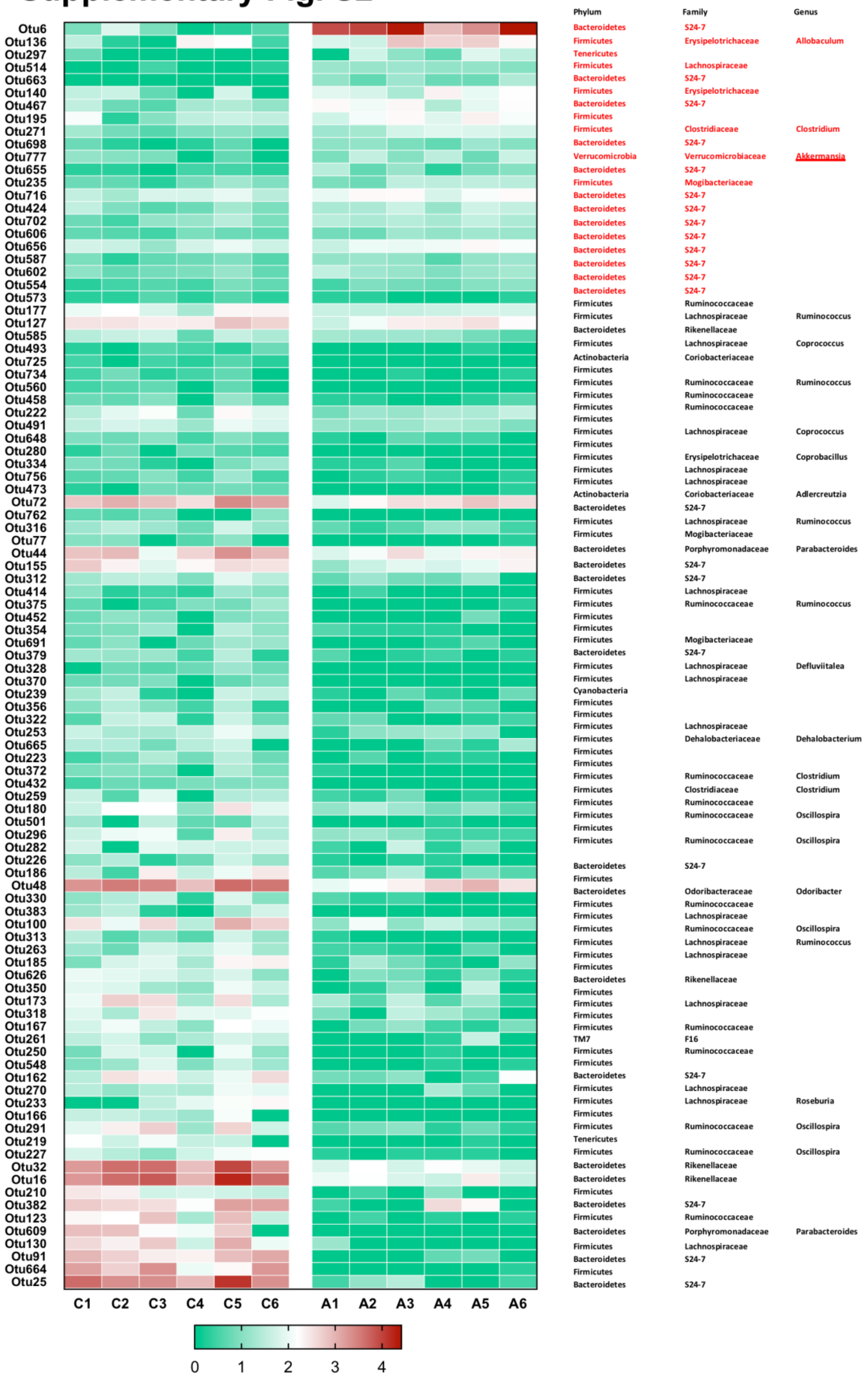

Supplementary Figure S2. Heatmap of gut bacterial profiles. OUT annotation of gut microbiota with significant difference between control (C) and andrographolide (A) groups. Control, db/db mice administered with vehicle; AG, db/db mice administered with 150 mg/kg andrographolide, n=6, **p* < 0.05 versus control group.
